# Vitamin D and Calcium Supplementation and Urolithiasis: A Controversial and Multifaceted Relationship

**DOI:** 10.3390/nu15071724

**Published:** 2023-03-31

**Authors:** Piergiorgio Messa, Giuseppe Castellano, Simone Vettoretti, Carlo Maria Alfieri, Domenico Giannese, Vincenzo Panichi, Adamasco Cupisti

**Affiliations:** 1 Nephrology, Dialysis and Renal Transplantation, Fondazione IRCCS Ca’ Granda Ospedale Policlinico Milan, 20122 Milan, Italy; 2 Department of Clinical Sciences and Community Health, University of Milan, 20122 Milan, Italy; 3 Department of Clinical and Experimental Medicine, University of Pisa, 56126 Pisa, Italy

**Keywords:** urolithiasis, vitamin D, calcium, dietary supplementation, chronic kidney disease

## Abstract

Patients with urolithiasis, and particularly those with hypercalciuria, frequently have a marked reduction of bone mineral content up to the levels of osteoporosis, with a significant increase in bone fracture risk. For these reasons, the indication to prescribe vitamin D and/or calcium supplementations is very frequent in such patients. On the other hand, both calcium supplementation, and even more vitamin D therapy, can worsen the risk of developing urolithiasis by increasing calcium, phosphate, and oxalate urinary excretion. Despite the clinical and practical relevance of this issue, the evidence on this topic is scarce and contradictory. Therefore, some concerns exist about how and whether to prescribe such supplements to a patient with a history of kidney stones. In this narrative review, we resume some pivotal pathophysiological concepts strictly related to the dealt topic, and we draw some considerations and personal opinions on the pros and cons of such prescriptions. Finally, we share with the reader our pragmatic algorithm for handling the urolithiasis risk in patients who have strong indications to be prescribed vitamin D and calcium supplementations.

## 1. Introduction

Urolithiasis (UL) is one of the most common diseases in the world and about one of every 10–12 subjects of the general population experiences an episode of UL, with a prevalence in males that is about double compared to females. Half or more of these subjects, when untreated, undergo at least one urinary stone recurrence in their lifetime [1,2,3,4].

Urolithiasis, particularly when relapsing and/or complicated by obstructive nephropathy or infection, can induce a renal damage which can evolve into chronic kidney disease (CKD) and, eventually, though in a minority of patients, end stage kidney disease (ESKD) [5,6,7,8,9]. Moreover, UL episodes have a significant impact on the quality of life of patients and are associated with a significant increase in health care costs, both direct (related to interventions) and indirect (loss of working days) [2,10,11].

Although the etiology of UL is still far from being completely clarified, both genetic and environmental factors have been claimed to contribute, at variable extent, to the occurrence of kidney stone formation [12].

It is also well recognized that the main pathogenic mechanisms underlying this pathological process are linked to the breakdown of the balance between the urinary concentration of substances promoting (e.g., calcium, oxalate, uric acid, cystine, drugs) and those inhibiting (e.g., citrate, magnesium) the urinary stone forming process. In fact, an increase of promoters and/or a reduction of inhibitors, often in association with a relatively low urine volume or changes in hydrogen ion concentration, can increase the risk of crystal formation in the urinary environment, the first step toward urinary stone occurrence [13,14,15,16].

Although most urinary calculi are composed of multiple molecules, calcium salts are by far the most represented among all components, since approximately 80% of urinary stones contain a calcium salt (predominantly calcium oxalate and to a lesser extent calcium phosphate) [17,18]. Furthermore, Hypercalciuria (HC) is the most frequently found metabolic abnormality in the urine of kidney stone former (SF) subjects [19,20,21].

## 2. Hypercalciuria and Urolithiasis

The link between HC and UL has its roots in studies dated about one century ago. In 1939, Flocks RH reported on increased levels of calcium in the urine of SF patients, supporting a direct role of increased urinary calcium concentration in the pathogenesis of UL [22]. From then onward, a growing interest in the research field has been witnessed to better define the mechanisms and pathogenetic factors of HC, also suggesting diagnostic methodologies, easily executable in the real world of clinical medicine.

The definition of which level of daily urinary calcium excretion should be considered the threshold of “normality” above which one can make a reliable diagnosis of HC continues to be a matter of controversy and debate [23,24,25,26]. Due to the nature of the present manuscript, mainly directed to discuss some clinical practical aspects, we do not think it is appropriate to discuss this topic here. We decided to take, as a pragmatic threshold for defining HC, a reference level of urinary calcium greater than 4 mg/Kg/d or 250 mg in females and 300 mg in males, per day, in subjects consuming a diet with a daily calcium content of 1000–1200 mg, which represents the recommendation of calcium intake for general population.

Classically, HC has been grouped into three main categories: intestinal (or absorptive) HC; renal leak HC; and bone resorptive HC [23,24,25]. However, the existence of clear pathogenetic differences among the above-listed types of HC, and, even more, the clinical relevance of this classification has been questioned. Although this topic might be of great physio-pathological and clinical importance, we do not consider it useful to go into the details in this discussion, limiting ourselves to dealing with some peculiar aspects which, in our opinion, are of greater practical interest in relation to the main purpose of this review.

### 2.1. Intestinal Absorptive HC

An increased entry of calcium from the intestine into the intracorporeal pool can occur through two different pathways: the first is mainly secondary to an increase in the amount of calcium intake from dietary sources and/or supplements, which induces an increased intestinal absorption through paracellular diffusive transport mechanisms mainly driven by concentration gradient; the second one is mainly due to the increased transcellular transfer of calcium through the enterocytes, by active saturable transport mechanisms highly dependent on vitamin D availability and effectiveness [26]. 

Given the shortage of balance studies, a precise estimation of the net amount of calcium absorbed through the intestinal route at the usual dietary intakes still remains only vaguely defined [27]. Nevertheless, one can expect to have an increased net intestinal calcium absorption either in the case of an increased absolute amount of its dietary content and/or from a more active intestinal calcium transport due to increased levels of or an augmented sensitivity to active vitamin D metabolites. There are, however, some unique differences between these two different types of absorptive HC, particularly regarding the associated risk of developing UL, which deserve to be specifically discussed.

First, given that a normal subject, under conditions of metabolic equilibrium, usually absorbs 15–20% of the calcium content of a normal diet (800–1000 mg of calcium), it is expected that the HC according to the pragmatic definition reported beforecan be observed only if the calcium content of the diet exceeds 1200–1500 mg. On the other hand, if an individual is exposed to higher levels of vitamin D and/or has increased sensitivity to vitamin D activity, the resulting increase in calcium absorption, via the active absorption pathway, may result in HC even with a dietary calcium content within or even below the recommended range.

**Figure 1:**
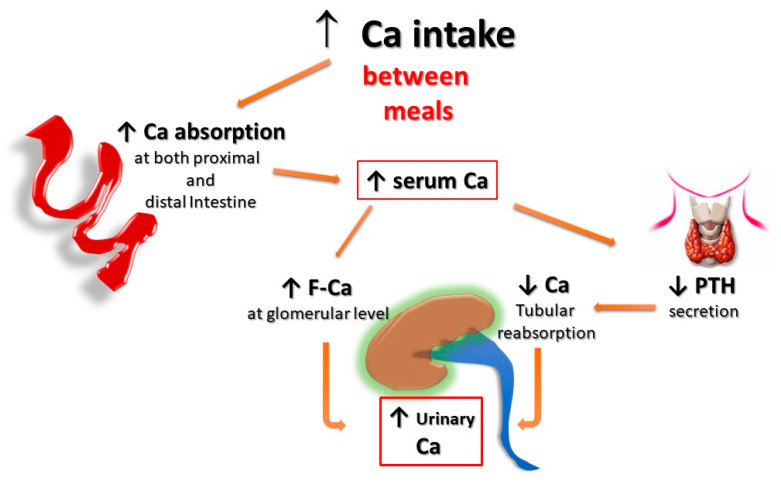
**Potential effects of Ca supplementation outside the meals on UL risk.** The amount of calcium absorbed is greater when calcium supplements are given outside meals, as calcium cannot bind to dietary anions (phosphate, oxalate, sulphate, etc.), and this leaves room for intestinal absorption an increased amount of both calcium and unbound anions, resulting in increased urinary excretion of calcium, oxalate, and phosphate. **F-Ca**: filtered Calcium; **PTH**: Parathyroid Hormone.

**Figure 2:**
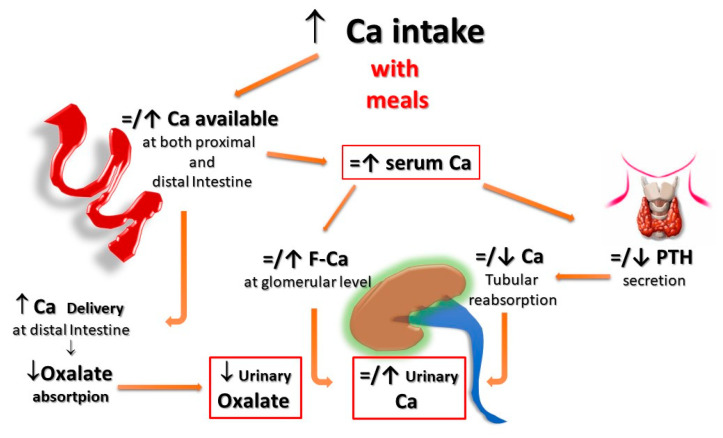
**Potential effects of Ca supplementation with meals on UL risk.** When calcium supplements are taken with meals, calcium can bind to dietary anions (phosphate, oxalate, sulphate, etc.), with a reduced absorption of calcium itself and the associated anions. Within the normal range of dietary calcium intake (800–1000 mg) and in the absence of elevated vitamin D levels, this could even translate into a reduction of the urinary lithogenic risk. **F-Ca**: filtered Calcium; **PTH**: Parathyroid Hormone.

Second, for the same amount of oral calcium introduced daily, the amount of calcium absorbed is much lower when the oral calcium intake is introduced primarily through the consumption of calcium-rich foods or beverages during meals compared to net calcium absorption following the intake of calcium supplements or calcium-rich beverages outside meals. This difference is secondary to the reduced bioavailability of calcium to be absorbed when introduced with food. This occurs because calcium ions can bind to dietary anions, namely phosphate, oxalate, sulphate, etc. This mechanism does not work when calcium is taken away from meals. This difference is of great relevance regarding the risk of UL as increasing calcium intake during meals could also play a protective role, given the potential binding of oxalate by calcium in the intestine, resulting in reduced intestinal absorption and urinary excretion of this potent lithiasis promoter (Figure 1 and Figure 2). Indeed, several decades ago, we demonstrated that SF patients who switched from a calcium-free diet to a calcium-restricted diet (400 mg/day) experienced a marked increase in their urine oxalate excretion and, despite a reduction in calcium excretion, the relative supersaturation of calcium oxalate nearly doubled [28]. In the same direction, even more indicative are the data of Curhan and collaborators who, through two prospective observational studies conducted on two large cohorts of subjects without a history of UL, clearly showed that the increase in calcium intake during meals was associated with a lower incidence of UL. In contrast, the increased calcium intake from calcium supplements, mainly consumed outside meals, appeared clearly associated with a higher incidence of kidney stones during the follow-up period [29,30].

**Figure 3:**
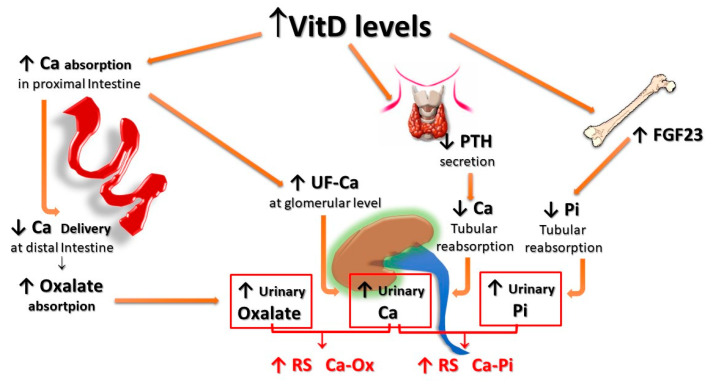
**Potential effects of vitamin D supplementation on UL risk.** Vitamin D stimulates active calcium transport, mainly in the proximal intestine, inducing HC even with calcium intake within the normal range; since less calcium reaches the distal gut, an increased amount of oxalate remains free for being absorbed in the distal tract; vitamin D also inhibits PTH production and stimulates FGF-23 production by osteocytes, with a resulting decrease of calcium and phosphate tubular reabsorption, respectively. All these effects translate into an increase in both the relative urinary supersaturations of calcium oxalate and calcium phosphate. **FGF-23**: Fibroblast Growth Factor-23; **F-Ca**: filtered Calcium; **HC**: Hypercalciuria; **PTH**: Parathyroid Hormone.

Finally, HC associated with increased vitamin D activity (due to both increased levels and/or increased sensitivity to its action) could impact even more relevantly on the risk of forming urinary stones. In fact, vitamin D not only enhances active intestinal calcium transport, inducing HC even with calcium intake within the normal range, but, since the vitamin D-mediated enhancement of calcium transport occurs predominantly, even if not exclusively, in the proximal gastrointestinal tract, a lower amount of calcium reaches the distal gut. Since it represents the major site of oxalate absorption, a decrease in calcium-bound oxalate and induces an increase in intestinal absorption and, thus, urinary excretion of oxalate. In addition, increased vitamin D activity induces decreased parathyroid hormone (PTH) and increased production and secretion of fibroblast growth factor-23 (FGF-23) by osteocytes, resulting in a decrease of calcium and phosphate re-absorption, respectively, at the distal and proximal renal tubular level, which, in turn, leads to increased urinary excretion of both calcium and phosphate. The overall final effect is an increase in the relative supersaturations of both calcium oxalate and calcium phosphate, contributing to an increased risk of calcium UL (Figure 3).

### 2.2. Bone Resorptive HC

Classically, the prototypical form of HC secondary to increased bone resorption is that associated with a primary increase in PTH secretion (namely, primary hyperparathyroidism, PHP), where the increased urinary calcium excretion is due to an increase in filtered calcium due to increased serum calcium concentration that is caused both by the increase in bone resorption, stimulated directly by PTH, and by the increase in intestinal calcium absorption secondary to the increase in calcitriol levels induced by PTH. The higher serum calcium levels result in increased glomerular filtered calcium, which exceeds the tubular calcium transport capacity, despite being potentiated by the action of PTH, and ultimately results in HC, which contributes to increased incidence of UL in patients with PHP [31]. In fact, this form of HC is of a mixed type, as both the absorption (intestinal) and the reabsorption (skeletal) components contribute to its occurrence.

Indeed, a PTH-independent form of increased bone resorption has been suggested as a frequent cause of HC in patients with urinary stones. This form of HC, often characterized by a high level of fasting urine calcium excretion (fasting HC), also given its many similarities to the calcium-losing form of HC, will be discussed in more detail in the next section.

### 2.3. Renal Leak HC

A renal tubular calcium leak, the so-called renal HC, has long been considered the second most common cause of HC, after intestinal HC [32,33]. Formerly, renal HC had been defined by the contemporary presence of increased excretion of calcium in fasting urine (fasting HC), normal to low calcium serum concentration, and normal to high parathyroid hormone (PTH) levels. All these characteristics are well suited to a condition of secondary hyperparathyroidism (SHP) as a compensation mechanism for renal calcium loss [34,35]. Apart from the rare forms of genetic defects involving renal tubular calcium transport [12,36,37], subsequent studies were not able to confirm the obvious presence of defects in any type of tubular calcium transport and, even less, the presence of SHP associated with fasting HC in SF subjects, suggesting that a PTH-independent increase in bone resorption could be the mechanism underlying the increase in urinary calcium excretion during fasting [38,39,40].

Among the pathogenetic mechanisms hypothesized to explain the PTH-independent increase in bone resorption associated with fasting HC, some authors have suggested that the increased production of some monocyte-derived cytokines could be implicated in osteoclast activation, with a consequent increase in mineral bone resorption [40,41,42,43].

It has also been suggested that consuming a diet rich in animal proteins, which is associated with an increased acid load mainly due to sulfate content, can directly increase bone resorption, due to the stimulating effect of hydrogen ions on osteoclast activity [44,45,46,47,48]. Furthermore, increased consumption of animal proteins has been reported to induce an increased production of prostaglandin E-2 (PGE-2) which is also a potent stimulator of bone resorptive mechanisms [49,50].

Other authors have suggested a role for some calcium sensor receptor (CaSR) polymorphic variants to explain the pathogenetic mechanism(s) of PTH-independent fasting HC [51]. In the past, when CaSR was firstly discovered and characterized [52], it was described as a G protein-coupled receptor, expressed on parathyroid cells, that acts mainly as a specialized sensor for extracellular calcium levels, whose activation induced by the increase in calcium concentration can effectively inhibit the secretion and synthesis of PTH. However, it was soon realized that CaSR is also expressed by several cells in many tissues other than the parathyroid glands, including renal tubular cells, where its activation is followed by decreased calcium uptake and thus increased urinary excretion [53,54]. Since the described CaSR polymorphic variant [51] was characterized by a gain of function of the receptor, the authors hypothesized that it could be a factor involved in the pathogenesis of PTH-independent fasting HC of SF patients where the rate of occurrence of this polymorphism was higher than in non-SF subjects.

Other genetic factors, such as polymorphisms in the soluble adenylate cyclase gene (ADCY10) or claudin-14 gene (CLDN14), have also been suggested to play a pathogenic role in the bone loss observed in patients with SF [14].

In addition to all these possible factors, there has often been added the false belief that it is considered appropriate and useful to advise all patients with urinary stones, especially those with HC, to follow a diet with reduced consumption of foods containing calcium.

Although this belief has been definitively disavowed by the results of scientific studies, it remains in the folds of popular cultures in many regions of the world, contributing to a condition of calcium deficiency which, far from being useful for counteracting the lithiasis process, certainly contributes to reducing bone mass.

In any case, whatever the pathogenic mechanism underlying the increase in fasting urinary calcium excretion, whether PTH dependent or not, many studies have reported that HC, particularly the fasting type of HC, is frequently associated with a reduced bone mineral content and an increased risk of bone fractures [55,56,57,58,59].

## 3. Indication to Vitamin D and/or Calcium Supplementation in the General Population

Skeletal fractures are one of the main health and social problems in the general population and low bone mass, particularly in the degree of osteoporosis, and it represents one of the main factors favoring and predisposing to skeletal fractures. Recent studies reported that osteoporosis, defined according to WHO as the presence of a bone mineral density (BMD) less than two and a half standard deviations (T ≤ −2.5) compared to values in young adults, is a pathological condition characterized by an increasing incidence worldwide [60,61]. 

Vitamin D has a well-recognized role in maintaining the skeletal system in a good health, thanks to its direct and indirect effects on mineral and bone metabolism, mediated by the interaction with its specific receptor (Vitamin D Receptor, VDR) expressed on the plasma membrane of the cells of some specific target organs. In fact, the interaction of vitamin D with the VDR expressed in the cells of bone tissue results in a global anabolic effect; vitamin D action at the intestinal level translates into an increased active transport of calcium from the intestinal lumen toward the blood, with an increase of intestinal calcium absorption and overall increased availability of this mineral for bone calcification process; meanwhile, the interaction of vitamin D with VDR expressed in the parathyroid cells is followed by the inhibition of the synthesis of PTH, downsizing the bone resorptive effects induced by this hormone [62,63,64].

However, in the last decades, a lot of studies clearly showed that VDR is expressed not only in the cells of the classic organs of vitamin D action (bone, intestine, parathyroid glands) but also in many other tissues [63]. As a consequence, in addition to the canonical effects of vitamin D on mineral and bone metabolism, it has also been suggested that vitamin D might have beneficial effects regards a number of other general pathological conditions. In fact, it has been widely reported by observational studies that low levels of vitamin D are associated with an increased risk of developing cardiovascular diseases, cancer, diabetes mellitus, infections, autoimmune and immune-related disorders, cardiovascular diseases (CVD), and even an increased mortality rate for any cause [65,66,67,68].

Furthermore, it has also been reported by a vast number of studies that vitamin D serum levels are often below the threshold of the concentration which has been defined by the Institute of Medicine as sufficient to avoid any related pathological effects [69,70].

For all these reasons, vitamin D, associated or not with calcium supplementation, has been more and more frequently prescribed to patients with low BMD, particularly in the osteoporosis range, and an increasing number of foods and/or beverages are enriched with vitamin D [71,72].

However, it is necessary to point out that, despite this widespread enthusiasm in suggesting an ever-wider use of vitamin D, motivated by these presumed positive pleiotropic effects, there is a complete lack of evidence to support these hypothetical advantages [73,74,75,76,77,78].

More importantly, the actual role of vitamin D and calcium supplementation on bone health and, in particular, on the possible reduction of the risk of fractures has long been the subject of controversy, given the very contradictory results of the data available so far [79,80,81,82,83]. Recently, a large-scale multicenter study questioned the usefulness of supplementation with vitamin D and/or calcium salts in preventing the risk of bone fractures in people with osteoporosis [84].

Anyway, as far as it concerns the UL risk, most of the clinical trials did not show any significant association between vitamin D and calcium supplementation and the risk of developing the urinary stone disease, at least in the general population. (This aspect will be discussed below).

## 4. Suggestions for Vitamin D and/or Calcium Supplementation in UL Patients

As previously discussed, the finding of low bone mass is particularly frequent in SF patients, particularly those presenting with HC [40,43,50,55,56,57,59]. Furthermore, it has been reported that subjects with a history of UL are particularly prone to sustaining bone fractures, with a fracture incidence reported over an observation period of up to 30 years almost four times higher than that observed in subjects without UL, matched for age and sex [58]. Additionally, vitamin D deficiency or insufficiency has been reported to be more frequent in UL patients than in the general population, with nearly 90% to 30% of SF patients having 25-OH-vit D3 levels lower than 30 and 12 ng/mL, respectively [85].

Since SF patients are also more prone to develop CV disease, it has also been claimed that vitamin D administration, thanks to its alleged CVD protective effects, could play a preventing role against this type of complication [86].

In addition, as if that were not enough, some experimental data suggested that inflammation, oxidative stress, and angiogenesis are involved in the development of the UL process. Hence, the putative anti-inflammatory, antioxidant, and antiangiogenetic activity of vitamin D could even help in preventing the lithogenic process itself [87,88].

Based on all these considerations, we can argue that SF subjects may be easily expected to be an ideal target for the prescription of vitamin D. 

On the other hand, we cannot forget that SF subjects, as previously discussed, are often characterized by increased calcium and oxalate excretion in their urine and that vitamin D and/or calcium supplementations can also concur to increase urinary calcium.

However, most, though not all, of the few randomized controlled trials reporting on the occurrence of UL events in subjects exposed to vitamin D and calcium supplementations gave an apparently reassuring message (Table 1): they claim no substantial difference in the occurrence of UL in the treated as compared with the untreated patients. 

However, it is worth making substantial considerations about some specific limitations of these studies [89,90,91,92].

**Table 1:** Characteristics and results of the main studies reporting on the effects of vitamin D, with or without calcium supplementation, on UL events.

Authors	Type of Study	Number and Characteristics of Patients	Type of Intervention	Study Duration	Bone-Related Outcomes	UL Events
Jackson RD *New Eng J Med* 2006 [92] Wallace RB et al. *Am J Clin Nutr* 2011 [93]	RCT	36,282 postmenopausal women aged 50–79 y	500 mg calcium carbonate plus 200 IU vitamin D3 twice daily (1000 mg and 400 IU daily, respectively), vs. a matching placebo	84 months	No significant difference in fracture rate	Significantly higher number of UL events in trated group
Malihi Z, et al. *Am J Clin Nutr* 2016 [84]	RCT	5108 participants; age 65.9 ± 8.3 y; females 41.9%; no history of UL	monthly 100,000 IU vitamin D3 supplementation vs. placebo	39 months	Not reported	No statistically significant difference in UL events between treated and placebo groups
Ferraro PM et al. *J Urol* 2017 [85]	Observational prospective study	HPFS: 51,529 male health professional; age 40–75 y	Divided into categories according to dietary vitamin D intake (<100, 100–199, 200–399, 400–599, 600–999, ≥1000 IU/day) and supplemental vitamin D (none, <400, 400–599, 600–999, ≥1000 IU/day)	HPFS: from 1986 to 2012	Not reported	HPFS: no association of dietary, or supplemental, or total vitamin D intake with UL occurrence
		NHS I: 121,700 female nurses; age 30–55 y		NHS I: from 1986 to 2012		NHS I: no association of dietary, or supplemental, or total vitamin D intake with UL occurrence
		NHS II: 116,430 female nurses; age 25–42 y		NHS II: from 1991 to 2011		NHS II: supplemental vitamin D intake, but not dietary, or total vitamin D intake, was associated to UL occurrence
Aspray TJ et al. *Am J Clin Nutr* 2019 [86]	RCT	379 adults aged ≥70 y (48% women; mean age: 75 y)	randomly allocated to 1 of 3 doses of vitamin D3 [12,000 international units (IU), 24,000 IU, or 48,000 IU] given once a month	12 months	Marginal changes in BMD at hip and FN; no difference between treatment groups	No UL event
Johnson KC et al. *Eur J Clin Nutr* 2022 [87]	RCT	2423 overweight/obese persons with prediabetes; age 60 ± 10 y women 44.8%	Daily 4000 IU of vitamin D_3_ vs. placebo	36 months	Not reported	No statistically significant difference in UL events between treated and placebo groups

Notes: BMD, bone mineral density; FN, femoral neck; HPFS, health professional follow-up study; IU, international unit; NHS, nurses’ health study; RCT, randomized controlled trial; UL, urolithiasis.

First, most of these studies have been carried out in elderly, mainly female subjects and these are not the hallmarks of a typical population of patients with UL who are usually young or middle-aged adults and more frequently males.

Second, the duration of all the studies that reported no difference in UL occurrence was lower than 3 years, so it cannot be excluded that with a longer observation period, some difference between treated and untreated cohorts could have been observed. 

Finally, it is not clear how many of the included patients had a history of previous UL events; so, it is quite arbitrary to draw any conclusion on what could be the risk of UL occurrence when such supplementations are prescribed to patients who already had one or more UL event, which represents the real medical problem in this clinical scenario.

## 5. Risks for Vitamin D and/or Calcium Supplementation in UL Patients

Based on the previous reported data, the rationale for fearing an increase in the risk of UL when vitamin D and/or calcium supplementations are prescribed to a patient with a history of kidney stones is quite strong, particularly in those patients with HC of any type.

In fact, calcium supplementation, particularly when given outside the meals, can increase the amount of calcium absorbed in the intestine, leaving more oxalate free for being absorbed. This results in both increased urinary excretion of calcium and a possible increased urinary oxalate (Figure 2). On the other hand, vitamin D supplementation can increase either calcium, oxalate, and phosphate intestinal absorption, with the consequent increased urinary excretion of all these three components and, hence, a potential increase in the relative saturation of both calcium-oxalate and calcium-phosphate salts (Figure 3).

This is reinforced by many experimental and observational data. We will limit ourselves to quoting just some more recent findings supporting the concept of the potential increased lithogenic risk associated with vitamin D and/or calcium supplementation.

An elegant experimental study was carried out in mice KO for the ABCC6 gene, which codifies for a transporter protein that increases the extracellular availability of pyrophosphate, one of the most potent inhibitors of ectopic calcification. The authors concluded that vitamin D and calcium supplementation accelerates the formation of Randall’s Plaque, which represent the starting point of the lithiasis process for calcium oxalate [93,94].

In an experimental study performed on 33 SF patients, who had 25(OH)D levels lower than 20 ng/mL, the supplementation with cholecalciferol, directed to effectively normalize 25(OH)VitD serum levels (form 11.8 ± 5.5 to 40.2 ± 12.2 ng/mL), resulted in a striking increase of urine supersaturation of both calcium oxalate and brushite [95]. 

A recent review and meta-analysis, which included 32 observational studies involving 23,228 participants, reported that SF subjects with HC have increased levels of circulating 1,25(OH)VitD3 and 25(OH)VitD3 compared with controls and SF subjects without HC [96].

Even more importantly, a large randomized controlled trial was designed for exploring the long-term (follow-up of 7 years) effects of vitamin D and calcium supplementations and carried out in 36,282 post-menopausal women. The results showed that the number of UL episodes was significantly higher in the group who received vitamin D and calcium supplementations as compared with the placebo-treated patients (Table 1) [80,97].

Another point that deserves particular attention is the reported susceptibility of some subjects to the toxic effects of vitamin D due to some genetic variants of genes involved in vitamin D metabolism or activity.

In their seminal study, Schlingmann and co-workers found recessive mutations in CYP24A1, the gene encoding 25-hydroxyvitamin D 24-hydroxylase, the key enzyme involved in the degradation of 1,25-dihydroxy vitamin D3, in six children with spontaneous hypercalcemia and also in a cohort of neonates in whom severe hypercalcemia developed after vitamin D administration [98]. Since then, many studies have reported a strong association between an increased risk of forming urinary stones and some genetic variants in the CYP24A1 gene, VDR gene polymorphisms, or other genetic variants of some specific solute transporters (SLC34A1, SLC34A3), suggesting that some individuals may be more prone than others to suffer from pro-lithogenic effects of vitamin D administration [99,100,101,102,103,104,105].

These new findings have prompted some colleagues to suggest that it could be appropriate to apply a nutrigenetic and nutrigenomics approach when prescribing a diet and/or supplements in UL patients [106].

Based on this pathophysiological background, many authors expressed their concern about prescribing such supplementations to SF patients, particularly for those who are also hypercalciuric [107,108,109].

## 6. Conclusive Remarks

Trying to draw some definitive conclusions on the safety of vitamin D and/or calcium supplementation within the cohort of kidney stone formers, we should first premise that there is no clear evidence that vitamin D and calcium supplementations are responsible for or free from potential negative effects on the risk of promoting the occurrence of UL in the general population.

The shortage of consistent and solid information on such a critical issue is even greater dealing with patients who have a history of UL, since these patients have been only occasionally included in most of the largest controlled trials carried out, despite their being frequent recipients of such prescriptions, given their high propensity to suffer from osteoporosis. 

One should also consider that, even if recent studies cast some doubt on the efficacy of vitamin D and calcium supplementation on bone health, it is not easy to accept not correcting severe deficiency of vitamin D, even in subjects with a history of UL and with a contemporary high risk for bone fractures. 

In these cases, it is a hard job for any doctor to balance the potential risk for the bone on the one hand and the risk for kidney stone formation on the other hand. Consequently, the question already raised by Sundeep Khosla more than ten years ago [110] on what the practical approach for these patients should be remains unanswered.

Without any presumption of wanting to give any guideline on this topic, we will limit ourselves to sharing with the reader our pragmatic approach to this issue. 

First, we consider the strength of the indications for prescribing vitamin D and calcium supplementation, which, in our opinion, are represented by a severe reduction of BMD (T-score < −2.5) and/or vitamin D levels lower than 15 ng/mL and/or chronic and severe malabsorptive diseases. 

If there are indications for treatment, we should evaluate in each subject the risk of developing UL or, if the patient is already a SF, his/her risk of recurrence, with the aid of the nomogram published by Rule and collaborators a few years ago [111].

**Figure 4:**
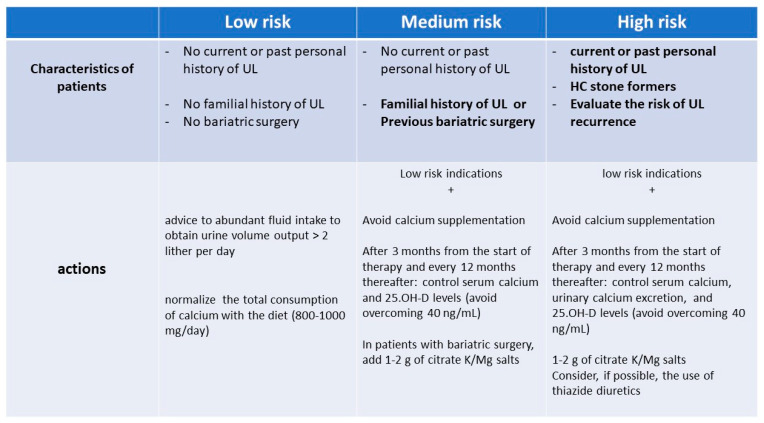
Definition of UL risk and suggested actions to be undertaken in subjects where there is a strong indication for prescribing vitamin D and/or calcium supplementation (see text).

Figure 4 illustrates our model of UL risk stratification of subjects in whom there is a strong indication to prescribe vitamin D and/or calcium supplements and the actions we suggest being taken.

Given the questionability of that pragmatic algorithm, we also suggest repeating this evaluation in each individual case over time, weighing the benefit–risk ratio. Namely, the effectiveness of the treatment on bone health parameters should be compared to safety parameters, in particular regarding the lithiasis risk, evaluating the changes in urinary calcium excretion and/or the appearance of hypercalcemia or a de novo occurrence or the relapse of lithiasis event.

Usually, at the end of most of the reviews on controversial topics, the authors suggest and wish for the start of dedicated trials. However, since we are well aware of the difficulties existing today for such studies to begin, we hope that a more systematic and shared collection of clinical follow-up data of patients with UL, submitted to treatments with supplementations of vitamin D and/or calcium, could be put into action, possibly sharing some preventive protocols. 

This could improve the knowledge of physicians in this field of clinical practice, hopefully improving, also, the efficacy of care of such patients.

## Data Availability

No original data were produced or used for the present manuscript.
